# Hysteria and Neurasthenia

**Published:** 1905-09

**Authors:** 


**Hysteria and Neurasthenia.** By J. MICHELL CLARKE, M.A., M.D. (Cantab.), F.R.C.P. Pp. ix., 298. London and New York: John Lane. 1905.

That the second number as well as the first of this series of Practitioners' Handbooks should have been entrusted to a local practitioner is a compliment to the Bristol School, but that the selection was amply justified is proved by the wholly admirable work which Dr. Michell Clarke has given us. The phenomena of hysteria have been known and separately recognised in a more or less perfect degree for long past, and good descriptions of the symptom-complex exist in our own and many other languages. The continued evolution of neurology, however, has made more extensive the area of what is now included under that conventional title, and has gone some way towards explaining the nature of the symptoms. Dr. Clarke, speaking from his own large experience and from his acquaintance with current medical literature, gives a satisfying account of all that is known for certain about this polymorphous malady. In accordance with the statements in the preface, he confines himself to the clinical side of the question, and avoids discussing the transcendental and even the theoretical points involved in the abstruse problem of the essential nature of hysteria. Though not in keeping with the general plan of the book, we should have been interested in learning more fully the views of so learned a neurologist as Dr. Clarke on the pathology of hysteria, of which one may truly say that up to the present *stat nominis umbra*. Upon the more practically important point of diagnosis, general and differential, Dr. Clarke may be studied with pleasure and profit, so ably has he handled this exceedingly difficult subject. In the matter of treatment, the most precise instructions are laid down for the management of these cases, which so often baffle both the medical attendant and friends of the patient.

Interesting as is the division upon Hysteria, we are, however, inclined to assign a higher value, if possible, to the section upon Neurasthenia. So recent has been the recognition of this ubiquitous disease, that we have no hesitation in saying that hitherto no really adequate and up-to-date account thereof has been published in English before the present delightful treatise. Such accounts as we have had provided for us are so marred with vagueness of expression as to leave a correspondingly vague picture in the mind of the reader, but no vagueness is apparent in Dr. Clarke's masterly exposition of the etiology, symptoms, and treatment of this newly-discovered old disease. We do not remember ever to have read any medical treatise with such plenary satisfaction, and would strenuously urge every practitioner to read it both for pleasure as well as instruction. The book appeals to all classes of practitioners alike, for the diseases herein treated, elusive and deceptive shadows, haunt the domains of the surgeon and gynæcologist hardly less than that of the physician.

The book is printed on good paper in clear type. There are many valuable illustrations, prepared from photographs of patients. The misprints are trivial and comparatively few, easily rectified in another edition. Dr. Clarke's vocabulary is extensive, his diction extraordinarily illuminating, his sentences well turned, lucid, and grammatical. The book has a good index, and is altogether meritorious in a high degree.

